# Vitamin D status and associations with substance use patterns among people with severe substance use disorders in Western Norway

**DOI:** 10.1038/s41598-022-17804-w

**Published:** 2022-08-11

**Authors:** Mitra Bemanian, Ranadip Chowdhury, Krister Stokke, Christer Frode Aas, Kjell Arne Johansson, Jørn Henrik Vold, Lars Thore Fadnes

**Affiliations:** 1 Bergen Addiction Research, Department of Addiction Medicine, Haukeland University Hospital, Post Box 1400, 5021 Bergen, Norway; 2 Department of Global Public Health and Primary Care, Faculty of Medicine, University of Bergen, Bergen, Norway; 3 Centre for Health Research and Development, Society for Applied Studies, New Delhi, India; 4 Division of Psychiatry, Haukeland University Hospital, Bergen, Norway

**Keywords:** Nutrition, Medical research

## Abstract

Chronic and harmful substance use is associated with a cluster of harms to health, including micronutrient deficiencies. Maintaining adequate levels of vitamin D is important for musculoskeletal and other aspects of health. In this prospective longitudinal cohort study, 666 participants drawn from outpatient opioid agonist therapy (OAT) clinics and community care clinics for substance use disorder in Western Norway were assessed annually for determination of serum 25-hydroxyvitamin D [s-25(OH)D] levels. Fifty-seven percent were deficient at baseline (s-25(OH)D < 50 nmol/l), and 19% were severely deficient (s-25(OH)D < 25 nmol/l). Among those deficient/severely deficient at baseline, 70% remained deficient/severely deficient at the last measurement (mean duration 714 days). Substance use patterns and dosage of opioids for OAT were not associated with vitamin D levels. One exception was found for cannabis, where consumption on a minimum weekly basis was associated with lower levels at baseline (mean difference: −5.2 nmol/l, 95% confidence interval [CI]: −9.1, − 1.3), but without clear time trends (mean change per year: 1.4 nmol/l, CI: − 0.86, 3.7). The high prevalence of sustained vitamin D deficiency in this cohort highlights the need for targeted monitoring and supplementation for this and similar at-risk populations.

## Introduction

Substance use disorder (SUD) contributes to 18 million healthy years of life lost annually worldwide, and opioids are the leading contributors to this adversity^1^. People with SUDs, including opioid use disorder (OUD), face many harms to health, one of which is poor nutrition and development of micronutrient malnutrition^2^. Micronutrient deficiencies are prevalent in SUD, in part owing to suboptimal diets and adverse dietary behaviors^3–5^. People with sustained and harmful substance use often experience economic difficulties and unstable housing, resulting in limited access to adequate nutrition^4,5^. Their intake of nutrient-dense foods, such as fresh produce, dairy, and sea food, is often limited and in many cases replaced by a high consumption of simple carbohydrates, high-sugar foods, and processed meats^4–7^. While enrollment in treatment programs for SUD, including opioid agonist therapy (OAT) for OUD, is known to improve anthropometric indices of nutritional status, adverse dietary patterns and micronutrient imbalances seem to persist^7–9^. Among the micronutrients, vitamin D is one that is well-studied, yet heavily debated; and it is one of the most frequently measured nutritional parameters in blood^10^. The current evidence on vitamin D status in SUD and OUD populations is conflicted. Whereas some studies report of poor vitamin D status among people with SUD^5^ and OUD^11^, others have found favorable levels among those with SUD compared to healthy controls^12,13^. Moreover, little is known about the substance-use or treatment-related risk factors of vitamin D deficiency in populations with severe SUD.

Vitamin D consists of a group of fat-soluble steroid-based prohormones essential for health through their role in the uptake of calcium and other micronutrients^14,15^. Vitamin D is available to humans in the form of vitamin D2 (ergocalciferol) and D3 (cholecalciferol) which are obtained from food ingestion or ultraviolet ray-induced dermal synthesis. Irrespective of source, vitamin D2 and D3 are converted to 25-hydroxyvitamin D [25(OH)D)] in the liver, which is converted in the kidneys to produce 1,25-dihydroxyvitamin D [1,25(OH)_2_D]—the biologically active metabolite of vitamin D^15^. Due to its structural stability and long half-time in circulation, serum or plasma concentration of 25(OH)D is the preferred indicator of vitamin D status^16^. The optimal blood concentration of vitamin D is debated in literature and integrally dependent on the metabolic needs of the individual, however there is a current consensus that serum 25(OH)D levels of 50 nmol/l or greater are representative of sufficient intake for most^17,18^. Vitamin D has an integral role in regulating calcium homeostasis and bone tissue turnover, and sustained and severe deficiency is known to cause rickets in children and osteomalacia in adults^19^. Beyond this, vitamin D is claimed to have an immunomodulating and neuroprotective role, and poor vitamin D status is associated with the onset and progression of autoimmune and neurodegenerative diseases including rheumatoid arthritis, multiple sclerosis and Parkinson disease^19,20^. Although inadequately studied, there is some preliminary evidence pointing to an association of poor vitamin D status with acquisition of SUD^21–23^.

Vitamin D deficiency could pose an additional, yet potentially mendable, burden to the health of people with sustained and severe substance use. The objective of this study was to assess the vitamin D status of an SUD, and primarily OUD, cohort drawn from outpatient clinics for OAT and community care clinics for SUD in Western Norway. We aimed to measure the prevalence of vitamin D deficiency in this population, and to assess to what extent deficiency was sustained through follow-up. Additionally, we aimed to determine whether factors related to substance use severity or OAT were associated with vitamin D levels in this population.

## Methods

### Study characteristics; design, population, data collection and study sample

This is a prospective longitudinal cohort study presenting data drawn from the multicenter INTRO-HCV and ATLAS4LAR studies^24,25^. The study was approved by the Regional Ethical Committee for Health Research, Norway (REK no: 155386 and REK Vest 2017/51), and conducted in accordance with the declaration of Helsinki and relevant guidelines and regulations. Participants provided written informed consent prior to enrolling in the study. Participants were recruited from a population of outpatients visiting OAT clinics or municipal community care clinics for SUD in Bergen and Stavanger, Norway. The cohort consisted mainly of individuals whose lives and health are severely impacted by SUD mostly with dependance to a range of substances including opioids, thus the descriptive term “*severe SUD*” is used. Participants were assessed yearly with a research nurse-led and questionnaire-based interview focused on somatic and mental health, psychosocial aspects and substance use patterns. Data was collected using the software CheckWare. Clinical data was obtained from the electronic medical record. The data presented was collected between March 2016 and June 2020 and includes a total of 1887 serum 25-hydroxy-vitamin D [25(OH)D] measurements from 666 participants. In total, 491 of the participants had at least two serum 25(OH)D assessments during the study period. Mean time elapsed between the first and last serum 25(OH)D assessment for participants with ≥ 2 blood samplings was 714 days.

### Measuring serum vitamin D; laboratory assays and definitions

Venous blood samples were collected and sent to the Department of Medical Biochemistry and Pharmacology at Haukeland University Hospital and the Department of Medical Biochemistry at Stavanger University Hospital (both accredited by ISO-standard 15189) for analysis of 25(OH)D concentration in serum samples. The former used a liquid chromatography-mass spectrometry (LC-MS) assay for assessment of serum 25(OH)D3 concentration for samples analyzed between March 2016 and November 2018, and thereafter the electrochemiluminescence immunoassay (ECLIA) was used to determine the pooled concentration of 25(OH)D2 and 25(OH)D3 (10% analytical variation). The latter laboratory used the LC-MS method for determining the pooled concentration of 25(OH)D2 and 25(OH)D3 (5% analytical variation). The difference in mean serum 25(OH)D in samples that were analyzed using these two methods was < 5 nmol/l. Data on the participants´ serum 25(OH)D concentration was obtained from the electronic medical record. The unit used was nanomoles per liter (nmol/l), and serum 25(OH)D levels ≥ 5 nmol/l were specified. In accordance with laboratory procedures the following cut-offs were used for describing vitamin D status: *deficiency* was defined as serum 25(OH)D < 50 nmol/l and *severe deficiency* was defined as serum 25(OH)D < 25 nmol/l^26,27^.

### Study variables; baseline, opioid agonist therapy, clinical and sociodemographic factors

Baseline was defined as the serum 25(OH)D measurement performed in closest proximity in time to the first annual health assessment for each individual. Subsequent serum 25(OH)D measurements were listed chronologically and included as follow-up, and time was defined as years from baseline. OAT was defined as receiving methadone or buprenorphine-based medication. We calculated an *OAT dose ratio* corresponding to the prescribed daily dose of medication divided by the expected mean dose (90 mg for methadone and 18 mg for buprenorphine) according to the World Health Organization^28^. As for the clinical factors, *injecting substances* was defined as having injected any substance within the prior 6 months. *Frequent substance consumption* was defined as consuming any of the following substances on a minimum weekly basis during the 12 months leading up to the annual health assessment: *alcohol*, *cannabis*, *benzodiazepines*, *stimulants* (amphetamine, methamphetamine, and cocaine), *non-OAT opioids* (e.g., heroin) and *tobacco* (smoking or consuming snuff products). Hepatitis C virus (HCV) status was determined by means of a quantitative polymerase chain reaction assay, where non-zero values were defined as *HCV infection*. Regarding sociodemographic factors, housing conditions for the last 30 days leading up to the annual health assessment were defined as *stable* (living in owned or rented home or at an institution) or *unstable* (being homeless, living at shelter or with friends and family). Source of income was presented as a dichotomous variable consisting of *paid labor* (full or part-time employment) and *receiving social benefits* (unemployment, disability or disease benefits and work assessment allowance)*.* Age was categorized into the following groups: < *30 years, 30–39 years, 40–49 years, 50–59 years and* ≥ *60 years.* Seasons were defined as *summer* (June–August), *autumn* (September–November), *winter* (December–February) and *spring* (March–May).

### Statistical analyses

Stata/SE 16.0 (Stata Corporation) was used for the generation of descriptive data and a linear mixed model. SPSS version 26.0 (IBM) was used for expectation maximization imputation. The software R version 4.0.3 (R foundation for Statistical Computing) with the package *mgcv* was used for the preparation of generalized additive models and their graphical presentations. The website Sankeymatic (*sankeymatic.com/build)* was used for the generation of a Sankey diagram. The threshold of statistical significance was set to *p* < 0.05 for all analyses. Nine percent of values were missing across the sociodemographic and clinical variables. These were assumed to be *missing and random* and expectation maximization imputation was performed to replace them with estimates. Descriptive data is presented with total numbers and percentages, only including valid values. Median vitamin D levels for different seasons are presented with interquartile range (IQR). The prevalence of *deficiency* and *severe deficiency* during different seasons of the year is presented with 95% confidence intervals. Generalized additive models and plots were generated to visualize the nonlinear associations of substance use severity with serum vitamin D concentration, adjusted for gender and age. To do this, a *substance use severity index*^29^ was generated based on the type, frequency and number of substances used (for details see Supplementary table S1). A generalized additive model of the association of weeks of the year with vitamin D concentration was generated to visualize the seasonality of vitamin D. A Sankey diagram was generated to display the flow between vitamin D status categories (i.e., vitamin D replete, deficiency and severe deficiency) between the first and last vitamin D assessment for participants with at least two measurements (*n* = 491). A linear mixed model was performed in order to estimate associations of clinical and sociodemographic factors with serum 25(OH)D concentration at baseline, as well as the time interactions of these associations. Clinical factors included *injection of substances*, *OAT dose ratio* and *frequent consumption of substances*. A variable for season was added to adjust for seasonal fluctuations in serum 25(OH)D concentration. The model was random intercept fixed slope with the estimator set to *restricted maximum likelihood*. Time was defined as years from baseline, and predictor variables were kept constant to baseline values. A time interaction was added to the variables *frequent substance consumption*, *injecting substances* and *OAT dose ratio* to investigate the impact of time on their associations with vitamin D. Partial adjusted models predicting associations of individual clinical factors with serum 25(OH)D concentration (adjusted for gender and age) are presented in addition to the adjusted model including all variables.

## Results

### Baseline characteristics of the cohort

The median age at baseline was 44 (IQR: 16), and 70% of participants were male (Table 1). Eighty-nine percent were enrolled in OAT, and of these 60% were prescribed buprenorphine-based medications and 39% were prescribed methadone. Twelve percent lived under unstable housing conditions and 53% had injected a substance at least once during the past six months. Frequent consumption of substances (on a minimum weekly basis) was reported by 77%, and the most common substances consumed were cannabis (50%) and benzodiazepines (38%), followed by alcohol (25%), stimulants (26%) and finally non-OAT opioids (14%).

**Table 1:** The table presents baseline characteristics of the cohort (N = 666).

Characteristic	n/N (%)
**Gender**	
Male	465/666 (70)
Female	201/666 (30)
**Age group**	
< 30 years	79/666 (11)
30–39 years	188/666 (28)
40–49 years	205/666 (31)
50–59 years	155/666 (23)
≥ 60 years	39/666 (6)
**Education level**	
Not completed primary school	36/666 (5)
Primary school (9 years)	300/666 (45)
High school (12 years)	267/666 (40)
≤ 3 years higher education	52/666 (8)
> 3 years higher education	11/666 (2)
**Source of income**	
Paid labor	50/666 (8)
Social benefits^1^	616/666 (92)
**Housing condition**^**2**^	
Unstable	79/666 (12)
Stable	587/666 (88)
**HCV infection**^**3**^	310/595 (52)
**Injecting substances**^**4**^	321/609 (53)
**Opioid agonist therapy**	594/666 (89)
Buprenorphine	356/594 (60)
Methadone	229/594 (39)
Other opioids	9/594 (2)
**Weekly substance use**^**5**^	
Alcohol	151/607 (25)
Cannabis	304/607 (50)
Stimulants^6^	159/607 (26)
Benzodiazepines	230/607 (38)
Non-OAT opioids	83/607 (14)
No weekly substance use	142/607 (23)
**Weekly tobacco use**^**7**^	564/607 (85)

^1^Social benefits include disability, disease and unemployment benefits and work assessment allowance.

^2^Stable housing included living in owned or rented housing or at an institution, unstable housing included homelessness, living at temporary camping sites or with friends or family.

^3^Hepatitis C virus infection, defined as non-zero values on a quantitative HCV-RNA assay at baseline.

^4^Self-reported injection of any substance during the 6 months prior to the first health assessment.

^5^Self-reported substance use on a minimum weekly basis during the 12 months prior to the first health assessment.

^6^Amphetamine, methamphetamine or cocaine, ^7^Self-reported tobacco use (smoking or snuff) on a minimum weekly basis during the 12 months prior to the first health assessment.

### Vitamin D status and seasonality

The median serum 25(OH)D concentration of the cohort was 45 nmol/l (IQR: 36) overall, ranging from 56 nmol/l (IQR: 37) in summer to 39 nmol/l (IQR: 34) in winter season (Table 2). Fifty-seven percent (CI: 54–59) had serum 25(OH)D levels < 50 nmol/l overall, ranging from 42% (CI: 40–50) in the summer season to 66% (CI: 58–67) in the winter and 66% (CI) in spring season. The corresponding numbers for serum 25(OH)D < 25 nmol/l were 19% (CI: 16–19) overall, 8.4% (CI: 7–13) in summer season and 27% (CI: 22–30) in winter season. Supplementary table S2 displays the association of serum 25(OH)D concentration with weeks of the year. The strongest positive associations were seen for week number 25–40, corresponding to mid-June to early October (summer and early autumn season in the Northern Hemisphere).

**Table 2:** The table presents median serum 25(OH)D levels and the prevalence of deficiency and severely deficiency for different seasons of the year.

	Total	Autumn	Winter	Spring	Summer
Vit. D, median (IQR), nmol/l	45 (36)	46 (39)	39 (34)	40 (30)	56 (37)
Deficiency, n/N (%)	380/666 (57)	96/180 (53)	106/160 (66)	114/172 (66)	64/154 (42)
Severe deficiency, n/N (%)	123/666 (19)	29/180 (16)	43/160 (27)	38/172 (22)	13/154 (8.4)

The table is based on the baseline measurement of all included participants (*n* = 666).

*IQR* 25–75 interquartile range; deficiency, serum 25(OH)D < 50 nmol/l; severe deficiency, serum 25(OH)D < 25 nmol/l.

### Changes in Vitamin D status over time

**Figure 1:**
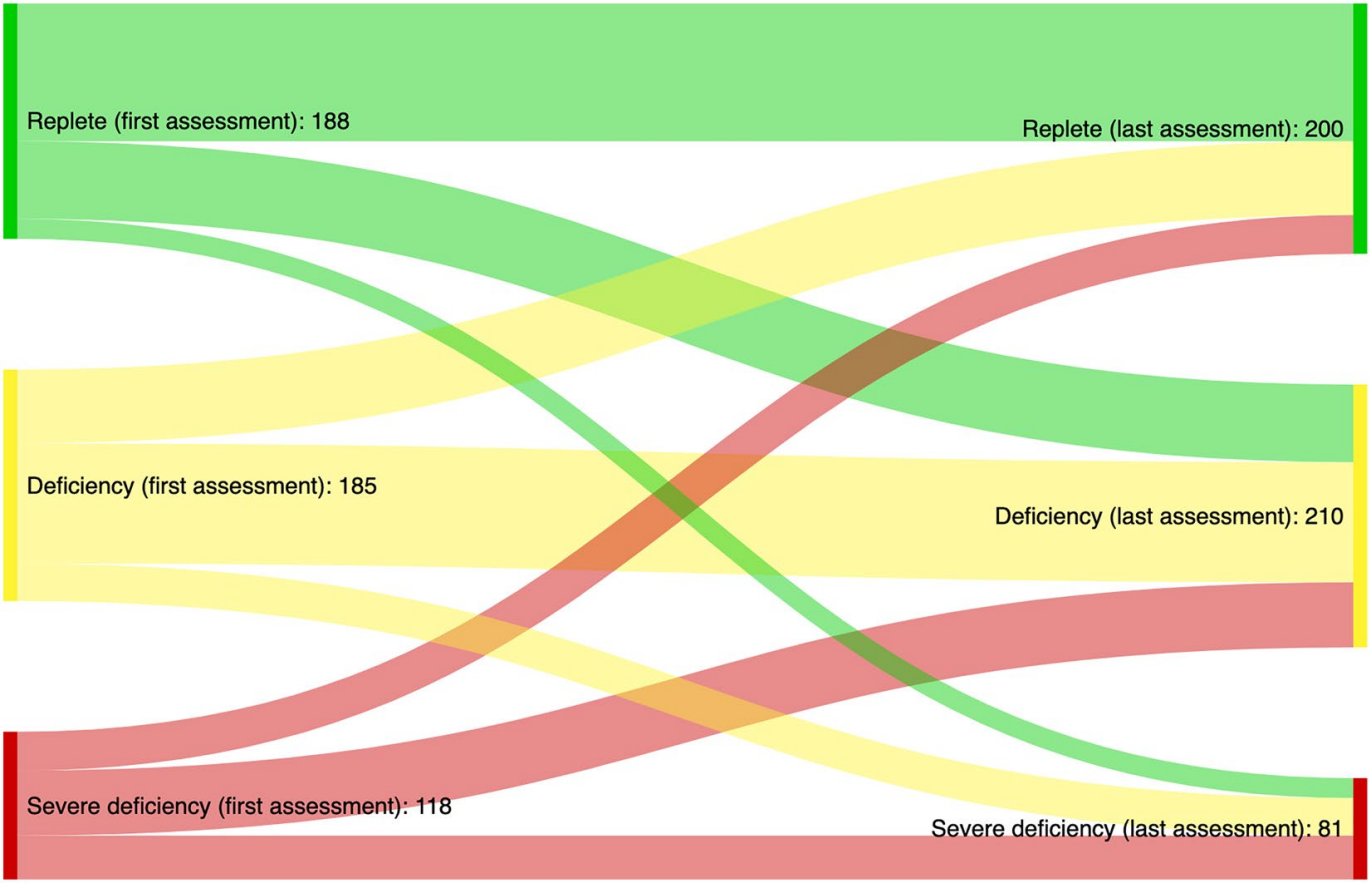
The figure displays changes in vitamin D status categories from the first (left) to the last (right) assessment for participants with at least two vitamin D measurements (*n* = 491). Definitions: vitamin D replete = serum 25(OH)D > 50 nmol/l, deficiency = serum 25(OH)D > 50 nmol/l severe deficiency = serum 25(OH)D < 25 nmol/l.

Among the 303 participants who were deficient or severely deficient at baseline, 213 (70%, CI: 65–75) remained deficient or severely deficient while 90 (30%, CI: 25–35) had sufficient levels at the last assessment (Fig. 1). Reversely, 78 of the 188 participants (41%, CI: 35–49) with sufficient levels at baseline had deficient or severely deficient levels at the last assessment and 110 (59%, CI: 51–65) remained vitamin D replete. The linear mixed model predicted a significant increase in serum 25(OH)D levels for the cohort over time (change per year: 5.19 nmol/l, CI: 0.33, 10.0) (Table 3).

**Table 3:** The table displays the results of a linear mixed model (restricted maximum likelihood regression) estimating associations of serum 25(OH)D concentration (nmol/l) with sociodemographic and clinical predictor variables at baseline (effect estimates), as well as the impact of predictors on changes in serum vitamin D concentrations over time (time trends per year).

	Partly adjusted*		Adjusted	
	Effect estimate	Time trend (per year)	effect estimate	Time trend (per year)
	Estimate (CI)	Slope (CI)	Estimate (CI)	Slope (CI)
**Serum 25(OH)D**			*57.2 (47.8, 66.6)*	5.19 (0.33, 10.0)
**Gender**				
Male			*0.00 (reference)*	
Female			0.81 (−2.73, 4.34)	
**Age**				
< 30			*0.00 (reference)*	
30–39			−1.57 (−6.43, 3.29)	
40–49			−2.50 (−7.60, 2.61)	
50–59			−3.15 (−8.55, 2.26)	
≥ 60			−0.62 (−8.42, 7.17)	
**Season**				
Summer			*0.00 (reference)*	
Autumn			*−6.06 (−8.89, −3.23)*	
Winter			*−11.4 (−14.4, −8.53)*	
Spring			*−10.8 (−13.7, −7.97)*	
**Source of income**				
Social benefits^1^	*0.00 (reference)*	*0.00 (reference)*	*0.00 (reference)*	*0.00 (reference)*
Paid labor	*8.73 (1.64, 15.8)*	0.42 (−3.99, 4.83)	*8.74 (1.52, 16.0)*	−1.52 (−6.10, 3.06)
**OAT dose ratio**^**2**^	−0.90 (−4.73, 2.94)	−0.11 (−2.61, 2.38)	−1.33 (−5.18, 2.52)	0.17 (−2.38, 2.72)
**Frequent consumtion**^**3**^				
Alcohol	0.85 (−3.50, 5.19)	−1.91 (−4.42, 0.59)	0.86 (−3.44, 5.16)	−1.62 (−4.18, 0.94)
Cannabis	*−4.88 (−8.63, −1.14)*	0.35 (−1.77, 2.47)	*−5.20 (−9.11, −1.29)*	1.41 (−0.86, 3.68)
Non−OAT opioids	1.94 (−3.62, 7.51)	−0.97 (−4.37, 2.43)	0.68 (−5.05, 6.40)	0.31 (−3.26, 3.87)
Stimulants^4^	−0.86 (−5.19, 3.46)	*−2.92 (−5.52, −0.32)*	−0.97 (−5.48, 3.53)	−2.17 (−4.94, 0.60)
Benzodiazepines	1.01 (−2.85, 4.88)	*−2.47 (−4.61, −0.33)*	3.47 (−0.70, 7.65)	−2.43 (−4.87, 0,01)
Tobacco^5^	0.40 (−7.01, 7.81)	−3.14 (−7.28, 1.00)	1.31 (−6.04, 8.65)	−3.82 (−8.01, 0.38)

Significant results are shown in italics (*p < *0.05). CI, 95% confidence interval.

*Adjusted for gender and age.

^1^Social benefits include disability, disease and unemployment benefits and work assessment allowance.

^2^The prescribed daily dosage of opioid agonist divided by the WHO mean recommended dosage (90 mg for methadone, 18 mg for buprenorphine). In this variable, zero represents no prescribed OAT medication.

^3^Self-reported consumption of a substance at a minimum weekly basis during the 12 months prior to the first assessment.

^4^Amphetamine, methamphetamine and cocaine.

^5^Self-reported tobacco use (smoking or snuff) on a minimum weekly basis during the 12 months prior to the first health assessment.

## Associations of substance use patterns with Vitamin D at baseline and over time

Lower serum 25(OH)D concentration at baseline was found for those with a frequent consumption of cannabis, compared to those with less or no use (mean difference: − 5.20 nmol/l, CI: − 9.11, − 1.29), however the time trend was not significant (mean change per year: 1.41 nmol/l, CI: − 0.86, 3.68) (Table 3). There were no significant associations with serum 25(OH)D levels at baseline or with changes in serum 25(OH)D levels over time for frequent consumption of alcohol, benzodiazepines, non-OAT opioids or stimulants. Similarly, no significant associations were found for the OAT dose ratio variable. A graphical representation of the non-linear association of serum 25(OH)D levels with the *substance use severity index* is presented in the as figure S3 in the Supplementary information. Substance use severity was not significantly associated with serum 25(OH)D concentration at baseline (*p* = 0.41). Due to strong correlations between frequent consumption of stimulants and injecting drug use (correlation coefficient = 0.42), injecting drug use was omitted from the linear mixed model. When including the variable for injecting substances in the model, similar findings were observed (see Supplementary table S4). An additional, sensitivity analysis with a linear mixed model of serum 25(OH)D concentration without the variable “source of income” provided relatively similar estimates as the model presented in Table 3 (see Supplementary table S5).

## Discussion

This study revealed an overall poor vitamin D status in this cohort drawn from an SUD, and predominantly OUD, population in Western Norway. Fifty-seven percent of the cohort had serum vitamin D concentrations below 50 nmol/l corresponding to deficiency, and 19% were severely deficient with serum levels below 25 nmol/l. Although vitamin D deficiency is not uncommon in Norway, the numbers presented in our study are markedly higher than those reported in studies on the general Norwegian population (57% vs. < 40%)^30–32^. In particular, the prevalence of severe deficiency is ~ 5 times higher in our study cohort compared to numbers presented in studies on the general Norwegian population (19% vs. ~ 4%)^30–32^. These finding are in line with another Norwegian study among heavy substance users reporting a mean serum vitamin D concentration of < 40 nmol/l, well below the threshold of deficiency^5^. Similarly, a descriptive study on methadone-users in the Boston-region of the US found that 36% were deficient in late summer-fall season^11^. In contrast, case–control studies from respectively Turkey and Australia have reported of more favorable serum concentrations of vitamin D among people with SUD and OUD compared to healthy controls^12,13^.

We did not find that substance use patterns, namely severity of substance use nor the consumption of most individual substance classes, were associated vitamin D levels. One exception was found for cannabis where frequent consumption was associated with lower vitamin D levels at baseline (however, without significant time trends). This finding is in line with another study describing lower serum vitamin D levels among heavy cannabis users compared to non-users^33^. Additionally, we did not find support of an association of opioid consumption with vitamin D levels in this population. Neither enrollment in OAT, the dose of opioid agonist prescribed, nor the use of non-OAT opioids were associated with vitamin D levels. The absence of consistent associations between substance use or treatment-related factors and vitamin D levels in our analyses suggests that there could be other factors contributing. A multitude of adversities are known to accompany sustained and severe substance use, including a high burden of disease, disability and economic disadvantage. In light of this, we found that the subset of participants in our cohort (8%) whose income stemmed from paid labor had markedly higher serum vitamin D levels compared to those receiving benefits. Employed individuals feasibly have a higher income and lower health impairment compared to the majority that rely on social benefits for income linked to disorders, disability, disease, or unemployment. The overall high prevalence of subjective health-complaints in this population, including fatigue^34^ and psychological complaints^35^ could contribute to behaviors that impact vitamin D status negatively, such as inactivity^36^ and less time spent outdoors exposed to ultraviolet radiation^37^.

Maintaining adequate vitamin D levels is important for many aspects of health^38^, and the high prevalence of deficiency poses a potential additional burden to the health of people with severe SUD. Addressing this could have positive outcomes on their health and well-being. In line with this, a randomized controlled trial on supplementation with 50,000 IU vitamin D every other week for 12 weeks showed promising results for several aspects of the health and well-being of people receiving methadone for OAT, including improved sleep quality, reduced psychological symptoms and blood markers of systemic inflammation and glucose tolerance^39^. As the prevalence of vitamin D deficiency, and in particular severe deficiency, was substantial in our study cohort, we suggest that the time has come to consider implementing targeted policies, including monitoring and/or supplementation in this and similar at-risk populations^40^.

### Strengths and limitations

Strengths of this study include its relatively large sample size among a “hard-to-reach” and “hard-to-treat” population. We managed to reach the majority of the target population, thereby minimizing the potential of a selection bias. Although participation in the study was offered to all eligible candidates, a possible minor selection bias related to sampling could be present due to the requirement of consenting in written form and in Norwegian language. This may have led to a skewed selection of participants with Norwegian language literacy, and thereby exclusion of some at-risk persons. A major strength of this study is its longitudinal design. Each participant, on average, had more than two successive vitamin D measurements. This allowed us to follow patterns in vitamin D and predictor variables over time, adding strength to the descriptive analyses and associations presented in this study. A limitation of this study is the absence of indicators of cerebral vitamin D status and variability (namely genetic variants of intracerebral 1, 25(OH)_2_D forming enzymes and effectors). A methodological limitation of this study is related timing of health assessments and blood samplings. Although health assessments were performed annually, the blood samples were not necessarily drawn in exact concurrence with these assessments. The two laboratories that analyzed serum samples did not employ identical techniques for determining 25(OH)D concentration over the entire study period, although the reference ranges as well as the measured levels were consistent. An important limitation on the interpretation of our analysis is related to the lack of information regarding the skin type of participants, as well as information regarding the adherence to treatment and treatment responses. As our design was an observational epidemiological study, confounders are a threat. Thus, identifying key confounders and adjusting properly for these is essential. It could be argued that our variable for economic conditions (i.e., source of income) was suboptimal. Some caution in interpretation of this is thus warranted, and we did also present a sensitivity analysis without this that was generally similar for other variables. Although associations between vitamin D and several different substance classes were assessed, most participants were people with opioid dependence enrolled in OAT. Our findings may therefore not be generalized to different SUDs or to those not receiving comparable treatment or follow-up. Additionally, our cohort is drawn from Western Norway in a northern latitude setting, and our findings are likely not generalizable to southern latitude settings.

## Conclusions

The vitamin D status of people visiting outpatient opioid agonist therapy clinics or community care clinics for SUD in Western Norway was inadequate. More than half were deficient and nearly a fifth were severely deficient. A majority of those deficient at baseline, remained deficient through follow-up. Frequent consumption of cannabis was associated with lower levels at baseline, but without consistent time trends. There were no clear associations for other individual substance classes, substance use severity or for the dosage of opioids consumed for opioid agonist therapy. These findings emphasize the need to address the vitamin D status of people with severe substance use disorders through targeted monitoring and/or supplementation. Further research is needed to establish more reliable causation of events.

## Supplementary Information

Supplementary Information.

## Data Availability

The datasets generated during and/or analyzed during the current study are not publicly available due to data protection requirements but are available from the corresponding author on reasonable request.
