# Supplementary material for: Vitamin D status and associations with substance use patterns among people with severe substance use disorders in Western Norway
Source: Sci Rep. 2022 Aug 11;12:13695. doi: 10.1038/s41598-022-17804-w (PMC9372185; doi:10.1038/s41598-022-17804-w)
Supplement: Supplementary file 1 — Supplementary Information. [file 41598_2022_17804_MOESM1_ESM.pdf]

Supplementary Information

Vitamin D deficiency among people with severe substance use disorders: a prospective cohort study from Norway

Mitra Bemanian, Ranadip Chowdhury, Krister Stokke, Christer Frode Aas, Kjell Arne Johansson, Jørn Henrik Vold, Lars Thore Fadnes

Table of contents

Supplementary Table S1: Calculation of the Substance Use Severity Index 2

Supplementary Figure S2: Association of Vitamin D concentration with weeks of the year 3

Supplementary Figure S3: Association of substance use severity with vitamin D concentration in serum 4

Supplementary Table S4: Linear Mixed Model of Serum 25(OH)D concentration (nmol/l) Adjusted for Sociodemographic and Clinical factors, Including Injection of Substances (Sensitivity Analysis) 5

Supplementary Table S5: Linear Mixed Model of Serum 25(OH)D concentration (nmol/l) Adjusted for Sociodemographic and Clinical factors, Omitting Source of Income (Sensitivity Analysis) 6

Supplementary Table S1: Calculation of the Substance Use Severity Index

	**Frequency of use**					
**Substance**	Never	< 1x/month	1-3x / month	1-3x/ week	4-6x/ week	Daily
Alcohol	0	1	2	3	4	5
Cannabis	0	1	2	3	4	5
Stimulants	0	1	2	3	4	5
Non-prescribed opioids	0	1	2	3	4	5
Benzodiazepines	0	1	2	3	4	5

Substance use severity score = sum of scores on each substance / 25 (min 0, max 1)

Supplementary Figure S2: Association of Vitamin D concentration with weeks of the year

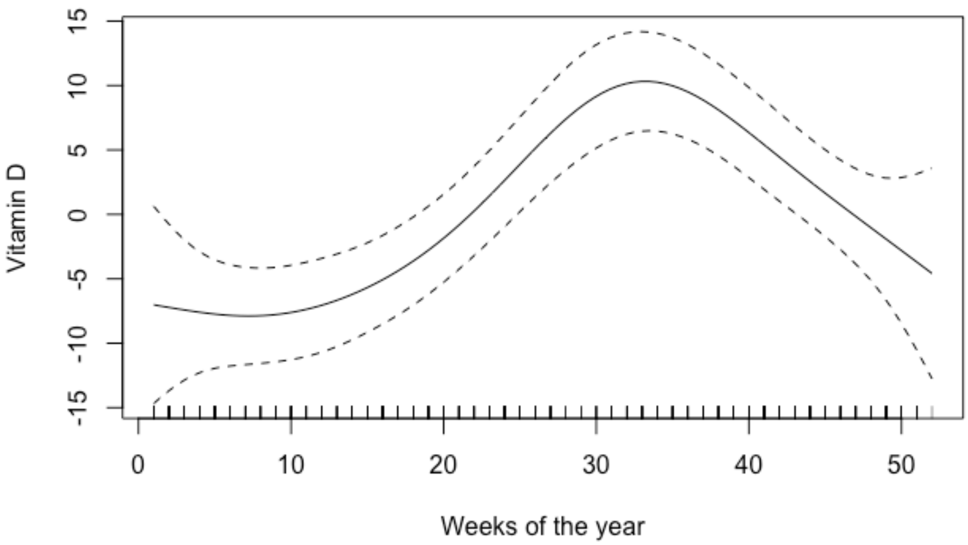

The graph was constructed using generalized additive models in R and shows associations between weeks of a year with vitamin D concentration in blood at baseline (n=666). The solid line depicts the association of s-25(OH)D concentrations with weeks of the year, and the area between the stippled lines represents the 95% confidence interval of this association.

Supplementary Figure S3: Association of substance use severity with vitamin D concentration in serum

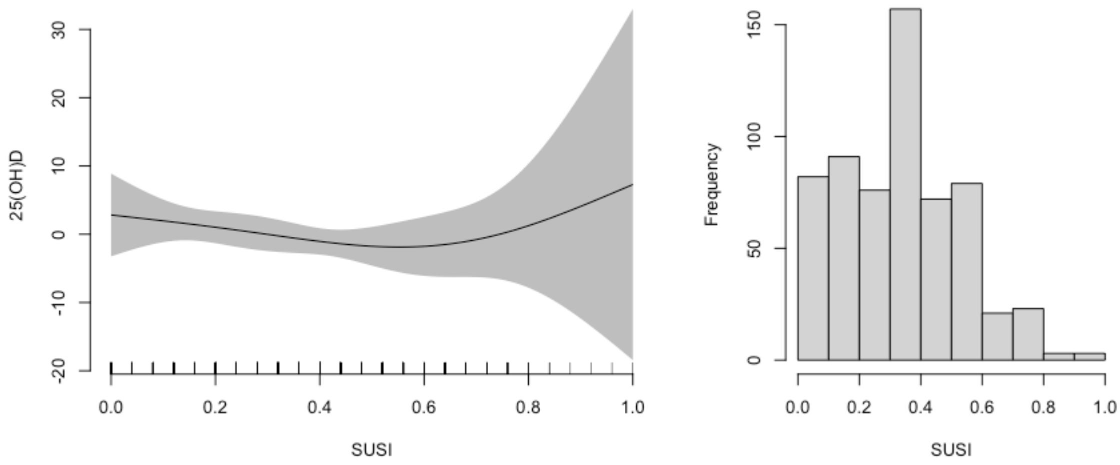

The figure to the left was constructed using generalized additive models in R and displays of the associations of serum 25(OH)D concentration with the substance use severity index (SUSI). The solid line depicts the association at various severity scores, whereas the shaded area represents the 95% confidence interval of this association. The figure to the right displays the distribution of substance use severity scores in the population.

Supplementary Table S4: Linear Mixed Model of Serum 25(OH)D concentration (nmol/l) Adjusted for Sociodemographic and Clinical factors, Including Injection of Substances (Sensitivity Analysis)

	**Partly adjusted[1]**		**Adjusted**	
	**Effect estimate**<br>Estimate (CI)	**Time trend (per year)**<br>Slope (CI)	**Effect estimate**<br>Estimate (CI)	**Time trend (per year)**<br>Slope (CI)
**Serum 25(OH)D**			*57.2 (47.8, 66.6)*	5.19 (0.33, 10.0)
**Gender**				
Male			*0.00 (reference)*	
Female			0.55 (-2.99, 4.08)	
**Age**				
< 30			*0.00 (reference)*	
30-39			-1.88 (-6.74, 2.98)	
40-49			-2.94 (-8.06, 2.18)	
50-59			-3.69 (-9.12, 1.73)	
≥ 60			-1.56 (-9.38, 6.25)	
**Season**				
Summer			*0.00 (reference)*	
Autumn			*-6.09 (-8.92,-3.27)*	
Winter			*-11.5 (-14.4,-8.57)*	
Spring			*-10.9 (-13.7,-8.00)*	
**Source of income**				
Benefits	*0.00 (reference)*	*0.00 (reference)*	*0.00 (reference)*	*0.00 (reference)*
Paid labor	*8.73 (1.64, 15.8)*	0.42 (-3.99, 4.83)	*8.32 (1.09, 15.5)*	0.02 (-2.55, 2.59)
**Injection of substances**	-3.21 (-7.01, 0.59)	-2.03 (-4.13, 0.08)	*-3.61 (-7.82, 0.60)*	-0.92 (-3.32, 1.48)
**OAT dose ratio[2]**	-0.90 (-4.73, 2.94)	-0.11 (-2.61, 2.38)	-3.61 (-7.82, 0.60)	-0.92 (-3.32, 1.45)
**Frequent consumtion[3]**				
Alcohol	0.85 (-3.50, 5.19)	-1.91 (-4.42, 0.59)	0.74 (-3.54. 5.03)	-1.54 (-4.10, 1.02)
Cannabis	*-4.88 (-8.63, -1.14)*	0.35 (-1.77, 2.47)	*-4.81 (-8.74,-0.88)*	1.53 (-0.75, 3.82)
Non-OAT opioids	1.94 (-3.62, 7.51)	-0.97 (-4.37, 2.43)	1.40 (-4.37,7.17)	0.50 (-3.09, 4.10)
Stimulants[3]	-0.86 (-5.19, 3.46)	*-2.92 (-5.52, -0.32)*	0.40 (-4.38, 5.18)	-1.81 (-4.74, 1.11)
Benzodiazepines	1.01(-2.85, 4.88)	*-2.47 (-4.61, -0.33)*	3.67 (-0.51, 7.84)	-2.37 (-4.81, 0.08)
Tobacco	0.40 (-7.01, 7.81)	-3.14 (-7.28, 1.00)	1,.0(-5.93, 8.74)	-3,49 (-7,71, 0.74)

The table displays the results of a linear mixed model (restricted maximum likelihood regression) estimating associations of serum 25(OH)D concentration (nmol/l) with sociodemographic and clinical predictor variables at baseline (effect estimates), as well as the impact of predictors on changes in serum vitamin D concentrations over time (time trends per year). Significant results are shown in italics.

CI, 95% confidence interval.

1. Adjusted for gender and age
2. The patients´ prescribed daily dose of opioid agonist divided by the WHO mean expected dose (90 mg for methadone, 18 mg for buprenorphine). In this variable, zero represents no prescribed OAT medication.
3. Self-reported injection of any substance during the 6 months prior to the first health assessment
4. Self-reported consumption of a substance at a minimum weekly basis during the 12 months prior to the first assessment
5. Amphetamine, methamphetamine and cocaine.

Supplementary Table S5: Linear Mixed Model of Serum 25(OH)D concentration (nmol/l) Adjusted for Sociodemographic and Clinical factors, Omitting Source of Income (Sensitivity Analysis)

	**Partly adjusted**[1]		**Adjusted**	
	**Effect estimate**	**Time trend (per year)**	**Effect estimate**	**Time trend (per year)**
	Estimate (CI)	Slope (CI)	Estimate (CI)	Slope (CI)
**Serum 25(OH)D**			*60.0 (53.8, 66.2)*	1.52 (-1.38, 4.42)
**Gender**				
Male			*0.00 (reference)*	
Female			0.63 (-2.91, 4.17)	
**Age**				
< 30			*0.00 (reference)*	
30-39			-1.74 (-6.61, 3.13)	
40-49			-2.84 (-7.96, 2.28)	
50-59			-3.84 (-9.25, 1.56)	
≥ 60			-0.39 (-8.09, 7.32)	
**Season**				
Summer			*0.00 (reference)*	
Autumn			*-6.10 (-8.93, -3.27)*	
Winter			*-11.29 (-14.19 -8.38)*	
Spring			*-10.73 (-13.59, -7.88)*	
**OAT dose ratio**[2]	-0.90 (-4.73, 2.94)	-0.11 (-2.61, 2.38)	-1.25 (-5.11, 2.60)	0.09 (-2.47, 2.65)
**Frequent consumtion**[3]				
Alcohol	0.84 (-3.51, 5.18)	-1.91 (-4.41, 0.59)	0.56 (-3.74, 4.86)	-1.34 (-3.88, 1.20)
Cannabis	*-4.93 (-8.67, -1.18)*	0.37 (-1.75, 2.49)	*-6.01 (-9.87, -2.15)*	1.41 (-0.83, 3.65)
Non-OAT opioids	1.95 (-3.62, 7.51)	-0.97 (-4.37, 2.43)	1.02 (-4.71, 6.76)	0.21 (-3.37, 3.78)
Stimulants[3]	-0.88 (-5.20, 3.44)	*-2.91 (-5.51, -0.32)*	-1.18 (-5.69, 3.32)	-2.30 (-5.07, 0.47)
Benzodiazepines	0.99 (-2.88, 4.85)	*-2.45 (-4.60, -0.31)*	2.85 (-1.30, 6.99)	-2.26 (-4.69, 0.16)

The table displays the results of a linear mixed model (restricted maximum likelihood regression) estimating associations of serum 25(OH)D concentration (nmol/l) with sociodemographic and clinical predictor variables at baseline (effect estimates), as well as the impact of predictors on changes in serum vitamin D concentrations over time (time trends per year). Significant results are shown in italics. CI, 95% confidence interval.

1. Adjusted for gender and age
2. The prescribed daily dosage of opioid agonist divided by the WHO mean recommended dosage (90 mg for methadone, 18 mg for buprenorphine). In this variable, zero represents no prescribed OAT medication.
3. Self-reported consumption of a substance at a minimum weekly basis during the 12 months prior to the first assessment
4. Amphetamine, methamphetamine and cocaine.
